# Comprehensive Analysis of Differentially Expressed lncRNAs in Gastric Cancer

**DOI:** 10.3389/fcell.2020.00557

**Published:** 2020-06-30

**Authors:** Nan Xiao, Yang Hu, Liran Juan

**Affiliations:** ^1^School of Life Sciences and Technology, Harbin Institute of Technology, Harbin, China; ^2^School of Pharmaceutical Sciences, Tsinghua University, Beijing, China

**Keywords:** gastric cancer, lncRNA, differential expression, coexpression network, survival biomarker

## Abstract

Gastric cancer (GC) is the fourth most common malignant tumor. The mechanism underlying GC occurrence and development remains unclear. Previous studies have indicated that long non-coding RNAs (lncRNAs) are significantly associated with gastric cancer, but a systematic understanding of the role of lncRNAs in gastric cancer is lacking. In recent years, with the development of next-generation sequencing technology, tens of thousands of lncRNAs have been discovered. However, a large number of unannotated lncRNAs remain unidentified in different tissues, including potential gastric cancer-related lncRNAs. In this study, RNA sequencing (RNA-seq) data from 16 samples of eight gastric cancer patients were obtained and analyzed. A total of 1,854 previously unannotated lncRNAs were identified by *ab initio* assembly, and 520 differentially expressed lncRNAs were validated in the TCGA expression dataset. Methylation and copy number variation (CNV) array data from the same sample were integrated in the analysis. Changes in DNA methylation levels and CNVs may be responsible for the differential expression of 91 lncRNAs. Differentially expressed lncRNAs were enriched in coexpressed clusters of genes related to functions such as cell signaling, cell cycle, immune response, metabolic processes, angiogenesis, and regulation of retinoic acid (RA) receptors. Finally, a differentially expressed lncRNA, AC004510.3, was identified as a potential biomarker for the prediction of the overall survival of gastric cancer patients.

## Introduction

Gastric cancer is the fourth most common malignant tumor in the world (Siegel et al., 2013). Early diagnosis and treatment are critical to improve the prognosis and reduce the mortality of patients with gastric cancer. Identifying important regulatory molecules in gastric cancer can provide valuable information for finding biomarkers for diagnosis and prognosis and for therapeutic targets.

In recent years, long non-coding RNAs (lncRNAs) have been shown to play a role in gene regulation. Abnormally expressed lncRNAs have been found in common malignant tumors, such as liver cancer and breast cancer (Eades et al., 2014; Amorim et al., 2016; Yang et al., 2017). These lncRNAs regulate tumor cells via various signaling pathways, such as Notch, mTOR, NF-κB, Wnt, and TGF-β, and play an important role in cell division, differentiation, apoptosis, invasion, and other processes (Song et al., 2013; Li et al., 2015; Cheng et al., 2019a). Many studies have shown that lncRNAs are also closely related to the occurrence, development, and metastasis of gastric cancer (Fang et al., 2015; Sun et al., 2016; Yang et al., 2016; Gu et al., 2017; Qi et al., 2019; Zhao et al., 2020). Compared with mRNA, lncRNA expression is more tissue specific (Tsoi et al., 2015; Cheng et al., 2018, 2019c; Cheng, 2019), which may provide new information for finding specific biomarkers for gastric cancer.

Long non-coding RNAs are involved in gastric cancer mechanisms by directly promoting or inhibiting cell growth, migration, invasion, or by regulating oncogenes and tumor suppressor genes (Fang et al., 2015; Tsoi et al., 2015; Yang et al., 2016; Cheng et al., 2019b). For example, H19 exhibits carcinogenic effects in gastric cancer. Yang et al. (2012) demonstrated that H19 is significantly increased in human gastric cancer tissues and AGS cell lines and accelerates gastric cancer cell proliferation. HOTAIR can promote the development of cancer by silencing the expression of miR-34a, inducing SNAIL, PI3K/Akt, and NF-κB signaling pathways (Liu Y. et al., 2015). Xu et al. found that FOXM and PVT form a positive feedback loop to promote gastric cancer proliferation and metastasis (Kong et al., 2015).

The expression of lncRNAs is related to the clinicopathological features of gastric cancer patients, such as TNM staging, tumor size and metastasis, differentiation, and infiltration (Wang et al., 2008, 2010; Sun et al., 2016; Han et al., 2019; Zou et al., 2019). Abnormally expressed lncRNAs can be used as biomarkers for the early diagnosis of gastric cancer. H19 is overexpressed in both gastric cancer and gastric cancer cell lines, and the same trend can be observed in plasma (Arita et al., 2013). AA174084 is less expressed in most gastric cancer tissues but is highly expressed in gastric juice and is associated with patient age, Borrmann typing, infiltration, and lymphatic metastasis (Shao et al., 2014). GACAT1 is significantly associated with lymph node metastasis and distant metastasis in gastric cancer tissues and may play an important role in gastric cancer metastasis (Sun et al., 2013).

Abnormally expressed lncRNAs can also be used to predict prognosis and monitor therapeutic response (Zeng et al., 2015, 2018; Zhao et al., 2015). The reduced expression of MEG3 in gastric cancer tissues is associated with TNM staging, depth of invasion, and tumor size and may indicate poor prognosis (Li et al., 2016). The overexpression of HOTAIR in gastric cancer is an effective predictor of tumor progression, associated with venous invasion, lymph node metastasis, and shortened survival in patients with diffuse gastric cancer (Liu et al., 2014). Hu Y. et al. (2014) found that GAPLINC is highly expressed in gastric cancer and can be used as a prognostic marker. The lower expression level of GAS5 in gastric cancer is also associated with poor prognosis (Sun et al., 2014). To obtain higher diagnostic accuracy and prognostic predictive effects, some studies have focused on combining multiple lncRNAs into a more efficient biomarker, such as the groups CTD-2616J11.14, RP1-90G24.10, RP11-150O12.3, RP11-1149O23.2, and MLK7-AS1 (Ren et al., 2016).

In summary, the molecular mechanisms of some individual gastric cancer-related lncRNAs have been discovered, but a systematic understanding of the relationship between lncRNAs and gastric cancer is still lacking. With the advance of high-throughput RNA sequencing (RNA-seq), the whole transcriptome, including lncRNAs, can be comprehensively discovered (Jiang et al., 2013; Zhao et al., 2017). More unannotated and tissue-specific lncRNAs can be identified by the *ab initio* assembly method (Wang et al., 2008; Iyer et al., 2015; Tang et al., 2017; Zeng et al., 2017). Further investigation and functional analysis are also essential for these novel lncRNAs.

In this paper, the GSE85467 dataset from the Gene Expression Omnibus (GEO), including RNA-seq, DNA-methylation array, and copy number variation (CNV) array data, was integrated and leveraged to study the potential function of lncRNAs in gastric cancer (Ooi et al., 2016). First, we aligned the RNA-seq data, assembled the transcriptome, and screened both annotated genes and the newly assembled lncRNAs (candidate lncRNAs) presented in the transcriptome. Next, we identified lncRNAs that were differentially expressed between gastric cancer and paracancerous tissues. The basic features of the candidate lncRNAs, such as transcript length, expression abundance, evolutionary conservation, and genomic location, were characterized and compared with the corresponding features of the annotated lncRNAs and protein-encoding genes in GENCODE v19. Then, we analyzed the factors that may affect the expression level of lncRNA and predicted the potential function of lncRNA in gastric cancer. Finally, we looked for possible biomarkers of gastric cancer prognosis.

## Results

### Candidate lncRNA Identification

In this paper, RNA-seq sequencing raw data (GSE85467) of eight gastric cancer tissues and paracancerous healthy tissues (16 samples) were downloaded from the NCBI GEO database. The dataset contained a total of 3,450,746,608 sequencing reads. Short sequences were aligned to the human reference genome GRCh37/hg19 by TopHat. The average mappable rate of the raw reads reached 77.78%.

A total of 92.6% of the 13,870 lncRNAs (23,898 transcripts) annotated in GENCODE v19 were detected in gastric cancer and paracancerous healthy tissue samples, which indicates that the sequencing depth of the RNA-seq dataset was sufficient for studying the function of lncRNAs in gastric cancer. This also suggests the correctness of the previous data processing steps, such as sequence alignment and transcript *ab initio* assembly.

**FIGURE 1:**
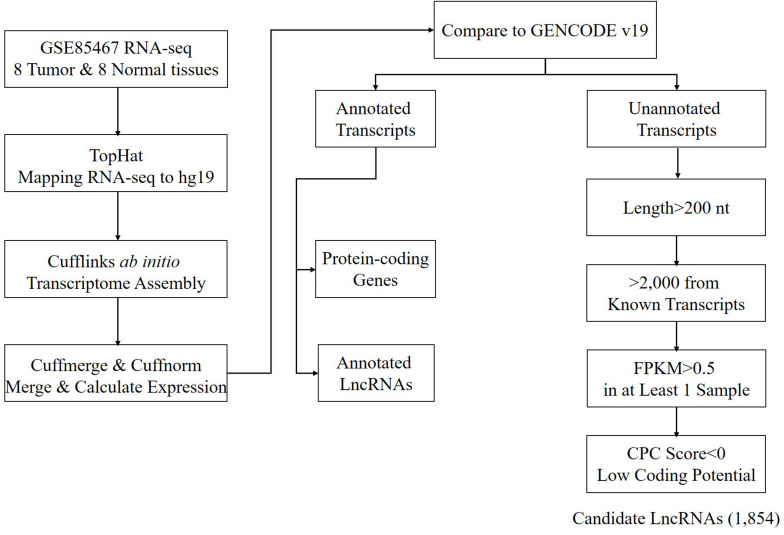
Workflow of candidate lncRNA screening.

Using the annotated non-coding RNAs and protein-coding genes in GENCODE v19 as a reference, a total of 1,854 newly assembled lncRNAs (2,039 transcripts) were screened out through a rigorous screening process (Figure 1 and Supplementary Table S1). The results of each step of the screening process are shown in Table 1. The novel lncRNAs were designated “candidate lncRNAs.” These candidate lncRNAs may be tissue-specific and function in gastric cancer tissues.

**TABLE 1:** Remaining transcripts after each filter step.

Step	Filters	Parameters	Number of transcripts	
1	Merged transcriptome	NA	327,364	
2	Length of transcripts	Length > 200 nt	24,836	
	GENCODE v19	No overlap		
	annotation			
3	Annotation by	Exon number	Monoexonic	Multiexonic
	exon number		19,169	5,667
4	Distance	Distance > 2,000 nt	17,928	
5	Expression	Max (FPKM) > 0.5	2,797	
6	Novel transcripts	NA	8,464	
7	CPC	Default	2,039	
Total number of newly assembled lncRNA transcripts			2,039	
			(1,854 lncRNA genes)	

### Basic Characteristics and Transcriptional Activity of the Candidate lncRNAs

Using the annotated non-coding RNAs and protein-coding genes in GENCODE v19 as a reference, a total of 1,854 newly assembled lncRNAs (2,039 transcripts) were screened out through a rigorous screening process (Figure 1 and Supplementary Table S1). The results of each step of the screening process are shown in Table 1. The novel lncRNAs were designated “candidate lncRNAs.” These candidate lncRNAs may be tissue-specific and function in gastric cancer tissues.

According to the location of the candidate lncRNAs relative to that of the protein-coding genes, the candidate lncRNAs were classified into four categories: 83.4% of the candidate lncRNAs were located in the intergenic regions and classified as intergenic lncRNAs; 15.2% of the candidate lncRNAs overlapped with the exons of the protein-coding genes or non-coding RNAs on the antisense strand and were classified as antisense lncRNAs; 1.3% of the candidate lncRNAs located in the same strand as the protein-coding gene within <2,000 nt, as cis-elements of the protein-coding gene, were classified as sense lncRNAs; only a small proportion (0.1%) of the candidate lncRNAs were completely located in the intron region of the protein-coding genes and were classified as intronic lncRNAs. The proportion of genomic location annotation of candidate lncRNAs was consistent with the statistical results in the literature.

**FIGURE 2:**
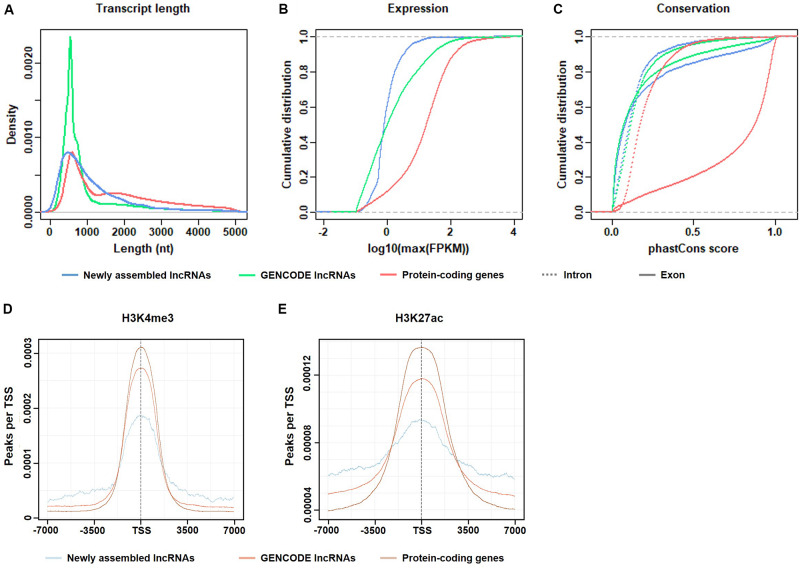
Basic features of the candidate lncRNAs. **(A)** Length distribution of the transcripts. **(B)** Maximum expression level (FPKM) of the transcripts. **(C)** Exonic/intronic conservation of the transcripts. **(D)** H3K4me3 enrichment near the TSS of candidate lncRNAs, GENCODE lncRNAs, and protein-coding genes. **(E)** H3K27ac enrichment near the TSS of candidate lncRNAs, GENCODE lncRNAs and protein-coding genes.

Basic features of candidate lncRNAs, such as transcript length, expression abundance, conservation of exon/intron regions, and the transcriptional activity marker H3K4me3 and H3K27ac signals, were characterized and compared to the annotated lncRNAs and protein-coding genes in GENCODE v19 (Figure 2).

As shown in Figure 2A, the transcript lengths of the candidate lncRNAs were slightly shorter than those of the protein-coding genes. In the candidate lncRNAs, the proportion of transcripts of 1,000–2,000 nt length was more than that of the GENCODE-annotated lncRNAs. The proportion of transcripts that were longer than 2,000 nt was similar, indicating that the RNA-seq depth was sufficient for *de novo* lncRNA assembly.

Figure 2B shows that the expression levels of the candidate lncRNAs were generally not high, which explains the possible reasons why these candidate lncRNAs were not found in previous studies.

Figure 2C shows that both the exon and intron regions of the candidate lncRNAs were less conserved than the corresponding regions of the protein-coding genes and were similar to the GENCODE-annotated lncRNAs. The conservation of exons was higher than that of introns. This result is logical and consistent with the results of previous studies.

In summary, the selected candidate lncRNAs were basically consistent with the characteristics of the known lncRNAs in GENCODE, which is consistent with the literature and biological principles, which indicates that the previously unexpressed lncRNAs identified in this paper are authentic and reliable.

To verify whether the candidate lncRNAs were transcribed in gastric cancer tissues, ChIP-seq data from the same gastric cancer tissues were used to capture the transcriptional activity signals near the transcription start sites (TSS).

As shown in Figures 2D,E, two epigenetic signals, H3K4me3 and H3K27ac, were enriched at the TSS in all transcripts, including candidate lncRNAs, GENCODE-annotated lncRNAs, and protein-coding genes. Both histone modifications were associated with transcriptional activation. The transcriptional activity signal intensity near the protein-coding gene TSS was the highest, and the signal intensity near the TSS of the candidate lncRNAs was lower than that of the GENCODE-annotated lncRNAs, which is consistent with the generally low expression level of the candidate lncRNAs (Figure 2B). This result indicates that in gastric cancer tissues, the transcriptional activity of these lncRNAs is regulated to different degrees.

### Differentially Expressed lncRNAs Between Cancerous and Normal Tissues

As shown in Figures 2D,E, two epigenetic signals, H3K4me3 and H3K27ac, were enriched at the TSS in all transcripts, including candidate lncRNAs, GENCODE-annotated lncRNAs, and protein-coding genes. Both histone modifications were associated with transcriptional activation. The transcriptional activity signal intensity near the protein-coding gene TSS was the highest, and the signal intensity near the TSS of the candidate lncRNAs was lower than that of the GENCODE-annotated lncRNAs, which is consistent with the generally low expression level of the candidate lncRNAs (Figure 2B). This result indicates that in gastric cancer tissues, the transcriptional activity of these lncRNAs is regulated to different degrees.

In this paper, differential expression analysis was carried out using the DEseq2 package in the R environment. This method is able to analyze candidate lncRNAs with relatively low expression. A total of 328 upregulated lncRNAs in gastric cancer tissues and 192 downregulated lncRNAs were found, of which 42 and 29 were candidate lncRNAs, respectively (Supplementary Table S2).

These differentially expressed lncRNAs may play an important role in the occurrence and development of gastric cancer. Table 2 lists the typical cases of differentially expressed GENCODE lncRNAs and candidate lncRNAs. Up/downregulated lncRNAs with | log2(fold_change)| > 3 and 5 smallest *p*-values were illustrated. Some of the differentially expressed lncRNAs in our results have also been reported in previous gastric cancer studies, such as PVT1 (ENSG00000249859.3), which is upregulated in gastric cancer tissues, promotes gastric cancer cell division and angiogenesis, and is associated with a poor prognosis (Kong et al., 2015).

**TABLE 2:** Typical up/downregulated lncRNAs in gastric cancer.

Gene ID	Gene symbol	Up/down	log2(fold_change)	*P*-value	q-value
ENSG00000269495.1	CTB-147C22.8	Up	7.379	1.63E−15	1.31E−11
ENSG00000128683.9	GAD1	Up	4.751	9.39E−12	3.36E−08
ENSG00000136231.9	IGF2BP3	Up	4.102	2.02E−11	6.50E−08
ENSG00000249550.2	RP11-438N16.1	Up	5.613	8.07E−11	2.16E−07
ENSG00000249859.3	PVT1	Up	3.433	5.39E−08	4.21E−05
ENSG00000233441.2	CYP2AB1P	Down	–5.272	6.49E−14	2.98E−10
ENSG00000139988.5	RDH12	Down	–3.857	4.74E−11	1.39E−07
ENSG00000100095.14	SEZ6L	Down	–3.220	3.98E−08	3.41E−05
ENSG00000264785.1	RP11-311F12.2	Down	–4.375	5.50E−08	4.21E−05
ENSG00000146151.8	HMGCLL1	Down	–3.180	7.64E−08	5.26E−05

**FIGURE 3:**
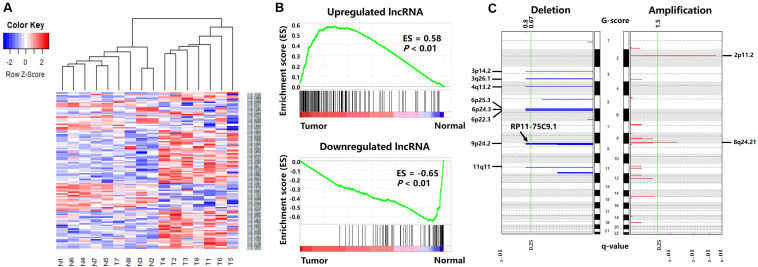
Differentially expressed lncRNAs between cancer and normal tissues. **(A)** Differentially expressed lncRNA clustering analysis. Rows are lncRNAs, and columns are samples. Blue denotes downregulation in gastric cancer and red denotes upregulation in gastric cancer. **(B)** GSEA enrichment analysis results. **(C)** Deletions and amplifications in gastric cancer genomes.

As shown in Figure 3A, hierarchical clustering analysis was performed on the differentially expressed lncRNAs. The heatmap illustrates that differentially expressed lncRNAs can accurately distinguish gastric cancer tissue samples from normal tissue samples, suggesting the potential role of these differentially expressed lncRNAs in the development and progression of gastric cancer.

To verify the reliability of the differentially expressed lncRNAs, we obtained gene expression data from 171 gastric cancer patients from the TCGA-STAD project, including 170 primary gastric cancer tissues and 20 paracancerous tissues. Then, we used GSEA (v 3.0) (Subramanian et al., 2005) to analyze the differentially expressed lncRNA enrichment in the TCGA gene expression data.

The results are shown in Figure 3B. The genes present in all TCGA samples were sorted according to the expression levels in the tumor and normal samples. When analyzing each gene of the TCGA expression dataset, if a gene was also a differentially expressed lncRNA in our result, the enrichment score (ES) increased, or vice versa. Figure 3B illustrates that the upregulated lncRNAs in the gastric cancer tissues were enriched in the high expression region of the TCGA tumor dataset, and the downregulated lncRNAs in the gastric cancer tissues were enriched in the high expression region of the TCGA normal dataset. This indicates that the differentially expressed lncRNAs screened in this paper also have extensive aberrant expression in TCGA gastric cancer samples.

To further explore the causes of the differential expression. We analyzed the DNA methylation data and CNV data from the same sample with RNA-seq data and investigated the possible genomic mechanism underlying the differential expression of the lncRNAs (Supplementary Table S3).

The CNV data were analyzed with the GISTIC algorithm. Two significantly amplified DNA regions and eight significantly deleted DNA regions (q-value < 0.25) were found in the gastric cancer genomes (Figure 3C). By comparing the genomic locations of the differentially expressed lncRNAs with the CNV regions, we found the lncRNA “*RP11-75C9.1*” located in a deletion region. The expression level of this lncRNA was downregulated in gastric cancer tissues. No differentially expressed lncRNA was found in the significantly amplified region.

Based on DNA methylation microarray data, we screened the DNA methylation sites located in the differentially expressed lncRNA promoter regions and calculated the Pearson correlation coefficient (pcc) between the methylation level and lncRNA expression level in all samples. We found that the expression level of 90 differentially expressed lncRNAs correlated with DNA methylation signals in the corresponding promoter region (pcc < -0.3). There were 56 upregulated lncRNAs and 34 downregulated lncRNAs, including two newly identified candidate lncRNAs. Typical cases are listed in Table 3.

**TABLE 3:** Potential DNA methylation-regulated lncRNAs in gastric cancer.

Gene ID	Gene symbol	Up/down	CpG	pcc
ENSG00000249859.3	PVT1	Up	cg05480350	–0.8624
ENSG00000196335.8	STK31	Up	cg14898779	–0.81715
ENSG00000248538.2	RP11-10A14.5	Up	cg24270182	–0.78429
ENSG00000185201.12	IFITM2	Up	cg09026253	–0.76839
ENSG00000018280.12	SLC11A1	Up	cg16926316	–0.76340
ENSG00000166573.4	GALR1	Down	cg04534765	–0.64735
ENSG00000152953.8	STK32B	Down	cg22551741	–0.567
ENSG00000234616.4	JRK	Down	cg06521562	–0.55163
ENSG00000133101.5	CCNA1	Down	cg05089090	–0.54299
ENSG00000257935.2	RP11-82C23.2	Down	cg20670075	–0.51918

### Functional Analysis of lncRNAs in Gastric Cancer

To investigate the functional role of lncRNA in the occurrence and development of gastric cancer, we constructed an mRNA–lncRNA coexpression network using the MCL algorithm. The network contained lncRNAs and protein-coding genes that were highly correlated at the expression level. The function of the protein-coding genes in each cluster was analyzed to predict the potential function of the lncRNAs.

All protein-coding genes and lncRNAs were clustered into 27 classes. Each class contained at least 45 genes. The whole network contained 6,734 protein-coding genes, 1,323 GENCODE-annotated lncRNAs, and 1,072 candidate lncRNAs, including 266 differentially expressed lncRNAs, forming 2,743,354 gene/lncRNA interactions.

For each cluster, GO analysis and KEGG pathway enrichment analysis were performed on the protein-coding genes. Then, the potential functions were assigned to the clusters, predicting the potential functions of the coexpressed lncRNAs in the clusters.

**FIGURE 4:**
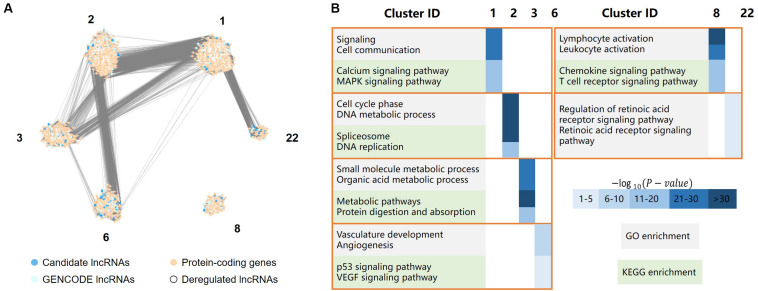
LncRNA functional analysis, including a total of 4,737 protein-coding genes, 701 GENCODE-annotated lncRNAs, and 413 candidate lncRNAs, the protein-coding genes and lncRNAs were clustered in six clusters. **(A)** The major clusters of the protein-coding gene/lncRNA coexpression network, where dark and light blue dots represent lncRNAs, light orange dots represent protein-coding genes, white dots represent deregulated lncRNAs. **(B)** GO/KEGG enrichment of the six major clusters, in which cluster 1 was mainly involved in cell signaling, cluster 2 was mainly involved in cell cycle processes, cluster 3 mainly related to the metabolic process in the stomach, cluster 6 was mainly involved in angiogenesis, cluster 8 was mainly involved in the immune response, and cluster 22 was primarily associated with the retinoic acid (RA) receptor signaling pathway.

Based on the GO and KEGG annotation, six clusters (clusters 1, 2, 3, 6, 8, and 22) may have functions related to the occurrence, development, and metastasis of gastric cancer (Figure 4). A total of 4,737 protein-coding genes, 701 GENCODE-annotated lncRNAs, and 413 candidate lncRNAs were in these six clusters, including 139 differentially expressed lncRNAs, accounting for 52.3% of all differentially expressed lncRNAs in the coexpression network.

Cluster 1 was mainly involved in cell signaling, such as the MAPK signaling and calcium ion pathways. The MAPK signaling pathway is critical for extracellular signal transduction into cellular responses and is involved in the development of cancer by regulating cell proliferation, differentiation, inflammation, and apoptosis (Zhang and Liu, 2002). The regulation of calcium signaling plays an important role in cell function. The dysregulation of the calcium signaling pathway leads to abnormal cell division, transcriptional processes, and cell death (Stewart et al., 2015), which are also associated with the development of gastric cancer.

Cluster 2 was mainly involved in cell cycle processes, such as DNA metabolism, DNA replication, and pathways associated with spliceosomes, which are related to the migration of gastric cancer cells.

Cluster 3 mainly related to the metabolic process in the stomach, such as the metabolism of organic acids, the metabolism of small molecules and the digestion and absorption of proteins.

Cluster 6 was mainly involved in angiogenesis, such as the vascular endothelial growth factor (VEGF) signaling pathway. Angiogenesis is critical for the development and metastasis of solid tumors (Folkman, 1990), and VEGF plays a central role in angiogenesis in cancerous diseases (Lazãr et al., 2008). The genes in this cluster were also significantly enriched in the p53 signaling pathway.

Cluster 8 was mainly involved in the immune response, such as the activation of leukocytes and chemokines and T cell receptor signaling pathways. The immune response may contribute to tumor development by influencing the infiltration and migration processes of tumor cells (Grivennikov et al., 2010).

Cluster 22 was primarily associated with the retinoic acid (RA) receptor signaling pathway. Previous studies have reported that RA is involved in many biological processes, such as cell growth, differentiation, and apoptosis (Hu K.-W. et al., 2014). The dysregulation of these processes is closely related to the occurrence and development of cancer. Changes in the RA signaling pathway are closely related to many inflammatory and neoplastic pathologies, including breast cancer, hematological malignancies, skin cancer, gastric cancer, and lung cancer (Poulain et al., 2009; McKenna, 2012; Ju et al., 2015; Liu Z.-M. et al., 2015). Previous studies have found that RA can reduce the viability of gastric cancer cells in a concentration-dependent manner (Patrad et al., 2018). It not only can significantly inhibit the proliferation of gastric cancer cells but also has a certain effect on the treatment of gastric cancer *in vivo* (Nguyen et al., 2016).

### Prognostic Biomarkers for Gastric Cancer

We further screened potential prognostic biomarkers for gastric cancer in the lncRNAs.

For each differentially expressed lncRNA, an expression cut-off value was set. Based on the cut-off value, the 170 patients from TCGA were divided into two groups: the high-expression group and the low-expression group. Then, the Kaplan–Meier 5-year survival analysis was performed based on the clinical information (current survival status, survival days before death) of the patients. A log rank test was conducted to analyze the significance.

**FIGURE 5:**
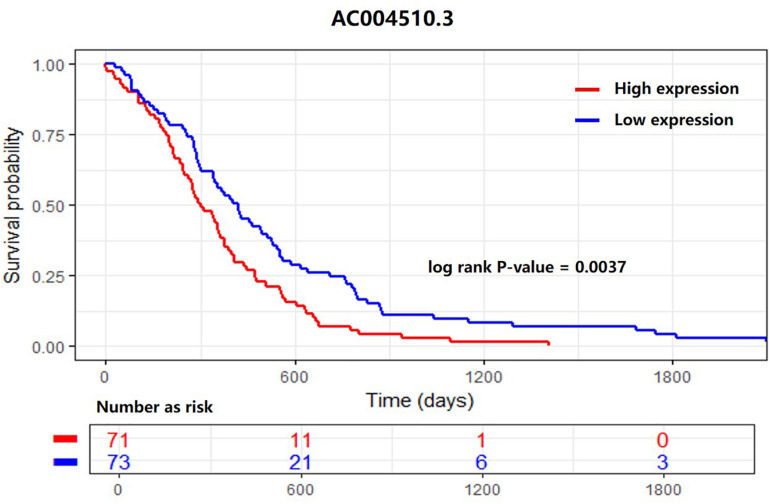
Kaplan–Meier survival analysis for the lncRNA AC004510.3, which is an intergenic lncRNA and is highly expressed in gastric cancer tissues. The lncRNA could significantly distinguish 170 patients into two groups with different 5-year survival statuses. The expression level was significantly correlated with the survival time of gastric cancer patients (*P*-value = 0.0037).

As shown in Figure 5, we found a differentially expressed lncRNA, AC004510.3 (ENSG00000267308.1) that could significantly distinguish 170 patients into two groups with different 5-year survival statuses. AC004510.3 is an intergenic lncRNA and is highly expressed in gastric cancer tissues. The expression level of AC004510.3 was significantly correlated with the survival time of gastric cancer patients (*P*-value = 0.0037). When the expression level of AC004510.3 in the gastric cancer tissues was higher than the cut-off value, the survival time of patients was significantly lower than that of patients with relatively low expression levels. This provides a possible prognosis prediction biomarker for gastric cancer patients.

## Materials and Methods

### Data Source

In this paper, we used (1) RNA-seq pair-end sequencing data from eight gastric cancer tissues and eight corresponding adjacent cancer tissues from the GEO database GSE85467 series (Ooi et al., 2016). The detailed sample and clinical information are described in Supplementary Tables S4, S5. (2) DNA methylation microarray data (GSE76153) were derived from the same sample as the sample used for the RNA-seq data. (3) The CNV microarray data (GSE85466) were derived from the same sample as the sample used the RNA-seq data. (4) The analyzed ChIP-seq data (GSE76153) in the GSE85467 series were also used, which are the H3K4me3 (GSM1975173) and H3K27ac (GSM1975175) histone modification data.

Moreover, the gene expression data of 170 gastric cancer tissues and 28 adjacent cancer tissues from 171 gastric cancer patients were downloaded from the National Cancer Institute (NCI) Genomic Data Commons (GDC) Data Portal. The data were generated by the TCGA-STAD project (Tomczak et al., 2015).

### RNA-Seq Data Processing

The raw RNA-seq data were aligned and assembled by Tophat (Trapnell et al., 2009) and Cufflinks (Trapnell et al., 2010). The RNA-seq reads were aligned to the human reference genome GRCh37/hg19 with GENCODE v19 annotation (Harrow et al., 2012). *Ab initio* assembly was conducted for each sample to identify previously unreported transcripts. Then, the transcriptomes of the 16 samples were integrated into a final transcriptome by the Cuffmerge component of the Cufflinks tool. Finally, the expression levels of both the protein-coding genes and lncRNAs were calculated by the Cuffnorm component of the Cufflinks tool.

### Candidate lncRNA Screening

Based on the GENCODE annotation, the transcripts present in the sample transcriptome were classified into three categories: protein-coding genes, annotated lncRNAs, and unannotated lncRNAs. The candidate lncRNAs were extracted from the unannotated lncRNAs by the following steps:

(1) Transcripts shorter than 200 nt or lacking strand information were removed.
(2) Transcripts overlapping annotated protein-coding genes or non-coding RNAs were removed.
(3) Mono-exon transcripts near (distance < 2,000 nt) annotated protein-coding genes or non-coding RNAs were removed.
(4) Mono-exon transcripts with low expression levels (FPKM < 0.5) in all samples were removed.
(5) Transcripts with high coding potential (CPC > 0) were removed. The coding potential was calculated using the Coding Potential Calculator (CPC) tool (Kong et al., 2007).

### ChIP-Seq Analysis

The corresponding ChIP-seq data (GSE76153) of two histone markers, H3K4me3 and H3K27ac, were used to check the transcriptional activity of the candidate lncRNAs. The “IRanges” and the “GenomicFeatures” (Lawrence et al., 2013) packages of the R environment were used to construct the histone modification peak and the TSS data. Then, we use the “ChipSeeker” (Yu et al., 2015) package to calculate and plot the signal intensities of the two histone modification signals around (up to 7,000 nt upstream and downstream) the TSS of each class of transcripts.

### Differential Expression Analysis of the lncRNAs

Differential expression analysis was conducted by the “DEseq2” (Love et al., 2014) package in the R environment. The criteria were the logarithmic change in expression level [|log2(fold-change)| > 1, *P*-value < 0.05, q-value < 0.05]. All transcripts in the integrated transcriptome were tested. Both protein-coding genes and lncRNAs that were differentially expressed between gastric cancer tissues and adjacent cancer tissues were identified.

### DNA Methylation and CNV Data Analysis

The β value of each CpG site in all samples was calculated using the “ChAMP” (Morris et al., 2013) package in the R environment to represent the methylation level. The methylation sites located in the differentially expressed lncRNA promoter regions (<2,000 nt upstream of the TSS) were selected. The pcc was calculated between the expression level and the corresponding DNA methylation signal of each lncRNA in all samples.

We used GISTIC 2.0 (Mermel et al., 2011) to determine significant DNA deletion or amplification.

### DNA Methylation and CNV Data Analysis

We employed a published method (Liao et al., 2011; Wang et al., 2018) to construct mRNA–lncRNA coexpression network. The network contained all differentially expressed protein-coding genes and lncRNAs, including both GENCODE-annotated genes and candidate lncRNAs. The detailed steps were as follows:

(1) For each lncRNA and protein-coding gene pair, we used the “WGCNA” (Langfelder and Horvath, 2008) package in the R environment to calculate the pcc of the expression levels of the gene pairs across all samples. The *p*-value was corrected using the Benjamin Hochberg method by the “multtest” package.
(2) Next, the genes with highly correlated expression levels were clustered by the Markov clustering (MCL) algorithm (Enright et al., 2002). The corrected *p*-value was used as the weight of the edge connecting each pair of genes.
(3) Cytoscape (Kohl et al., 2011) was used to visualize the clustering result.

### GO and KEGG Pathway Analysis

WebGestalt (Zhang et al., 2005)^1^ was used to perform GO/KEGG pathway enrichment analyses for all protein-coding genes present in each coexpression network cluster.

### Survival Analysis

We used the “survival” package in the R environment to perform the Kaplan–Meier analysis (Efron, 1988). The statistical significance of the results was analyzed by log rank test.

## Discussion

To explore the potential role of lncRNAs in the development and metastasis of gastric cancer, RNA-seq, ChIP-seq, CNV chip, and DNA methylation ChIP data from 16 gastric cancer and adjacent healthy tissue samples were obtained. A series of analyses were carried out at the genomic and epigenetic levels.

In the 16 gastric cancer and paracancerous tissues, we discovered 1,854 lncRNAs that had not been previously annotated. By characterizing the transcript length, expression abundance, conservation, and transcriptional activity, we showed that the basic features of the candidate lncRNAs screened in this paper were similar to the annotated lncRNAs and thus were reliable.

We analyzed the differentially expressed genes in the gastric cancer tissues and adjacent healthy tissue samples. A total of 328 upregulated lncRNAs and 192 downregulated lncRNAs were screened in the gastric cancer tissues, of which 42 and 29 were candidate lncRNAs, respectively. The result of GSEA enrichment analysis indicated that the differentially expressed lncRNAs found in this study were also abnormally expressed in the gastric cancer tissues of the TCGA patients.

By analyzing the DNA methylation and CNV data of the same sample, it was found that the expression levels of some differentially expressed lncRNAs were significantly correlated with the DNA methylation signals on their promoter region. We also found a differentially expressed lncRNA located in the CNV region. These results suggest possible causes of the aberrant expression of lncRNAs in gastric cancer.

We used the mRNA–lncRNA coexpression network to predict the functions of differentially expressed lncRNAs and found that the differential expression of lncRNAs may affect the occurrence and metastasis of gastric cancer via various biological processes, such as cell signaling, cell cycle, immune response, metabolic processes, angiogenesis, and RA receptor regulation.

Finally, we found that the lncRNA *AC004510.3*, which was upregulated in gastric cancer tissues, was significantly correlated with the expression level of gastric cancer tissues and the survival time of the patients. Thus, it has the potential to become a prognostic biomarker for gastric cancer patients.

The pathogenesis of gastric cancer remains unclear. Most patients with gastric cancer often have no symptoms at an early stage and are not diagnosed until the late stage, missing the best treatment period. The results of this paper have deepened the systematic understanding of the function and role of lncRNAs in the occurrence and metastasis of gastric cancer. We hope these results will provide novel information regarding gastric cancer.

## Data Availability Statement

All datasets presented in this study are included in the article/Supplementary Material.

## Author Contributions

LJ and NX designed the study. NX completed the experiment and coding. LJ, NX, and YH wrote the manuscript.

## Conflict of Interest

The authors declare that the research was conducted in the absence of any commercial or financial relationships that could be construed as a potential conflict of interest.

## Footnotes

1 http://www.webgestalt.org/

## References

[B1] Amorim M. Salta S. Henrique R. Jerónimo C. (2016). Decoding the usefulness of non-coding RNAs as breast cancer markers. *J. Transl. Med.* 14:265. 10.1186/s12967-016-1025-3 PMC5024523 27629831

[B2] Arita T. Ichikawa D. Konishi H. Komatsu S. Shiozaki A. Shoda K. (2013). Circulating long non-coding RNAs in plasma of patients with gastric cancer. *Anticancer Res.* 33 3185–3193. 23898077

[B3] Cheng L. (2019). Computational and biological methods for gene therapy. *Curr. Gene Ther.* 19:210. 10.2174/156652321904191022113307 31762421

[B4] Cheng L. Qi C. Zhuang H. Fu T. Zhang X. (2019a). gutMDisorder: a comprehensive database for dysbiosis of the gut microbiota in disorders and interventions. *Nucleic Acids Res.* 48 D554–D560. 10.1093/nar/gkz843 31584099 PMC6943049

[B5] Cheng L. Wang P. Tian R. Wang S. Guo Q. Luo M. (2019b). LncRNA2Target v2.0: a comprehensive database for target genes of lncRNAs in human and mouse. *Nucleic Acids Res.* 47 D140–D144. 10.1093/nar/gky1051 30380072 PMC6323902

[B6] Cheng L. Zhao H. Wang P. Zhou W. Luo M. Li T. (2019c). computational methods for identifying similar diseases. *Mol. Ther. Nucleic Acids* 18 590–604. 10.1016/j.omtn.2019.09.019 31678735 PMC6838934

[B7] Cheng L. Zhuang H. Yang S. Jiang H. Wang S. Zhang J. (2018). Exposing the causal effect of C-reactive protein on the risk of type 2 diabetes mellitus: a mendelian randomization study. *Front. Genet.* 9:657 10.3389/fgene.2018.00657 30619477 PMC6306438

[B8] Eades G. Zhang Y.-S. Li Q.-L. Xia J.-X. Yao Y. Zhou Q. (2014). Long non-coding RNAs in stem cells and cancer. *World J. Clin. Oncol.* 5 134–141. 24829860 10.5306/wjco.v5.i2.134 PMC4014785

[B9] Efron B. (1988). Logistic regression, survival analysis, and the Kaplan-Meier curve. *J. Am. Stat Assoc.* 83 414–425. 10.1080/01621459.1988.10478612

[B10] Enright A. J. Van Dongen S. Ouzounis C. A. (2002). An efficient algorithm for large-scale detection of protein families. *Nucleic Acids Res.* 30 1575–1584. 10.1093/nar/30.7.1575 11917018 PMC101833

[B11] Fang X.-Y. Pan H.-F. Leng R.-X. Ye D.-Q. (2015). Long noncoding RNAs: novel insights into gastric cancer. *Cancer Lett.* 356 357–366. 10.1016/j.canlet.2014.11.005 25444905

[B12] Folkman J. (1990). What is the evidence that tumors are angiogenesis dependent? *JNCI* 82 4–7. 10.1093/jnci/82.1.4 1688381

[B13] Grivennikov S. I. Greten F. R. Karin M. (2010). Immunity, inflammation, and cancer. *Cell* 140 883–899. 20303878 10.1016/j.cell.2010.01.025 PMC2866629

[B14] Gu J. Li Y. Fan L. Zhao Q. Tan B. Hua K. (2017). Identification of aberrantly expressed long non-coding RNAs in stomach adenocarcinoma. *Oncotarget* 8 49201–49216. 10.18632/oncotarget.17329 28484081 PMC5564761

[B15] Han J. Han X. Kong Q. Cheng L. (2019). psSubpathway: a software package for flexible identification of phenotype-specific subpathways in cancer progression. *Bioinformatics* 36 2303–2305. 10.1093/bioinformatics/btz894 31821408

[B16] Harrow J. Frankish A. Gonzalez J. M. Tapanari E. Diekhans M. Kokocinski F. (2012). GENCODE: the reference human genome annotation for The ENCODE Project. *Genome Res.* 22 1760–1774. 10.1101/gr.135350.111 22955987 PMC3431492

[B17] Hu K.-W. Pan X.-H. Chen F.-H. Qin R. Wu L.-M. Zhu H.-G. (2014). A novel retinoic acid analog, 4-amino-2-trifluoromethyl-phenyl retinate, inhibits gastric cancer cell growth. *Int. J. Mol. Med.* 33 415–422. 10.3892/ijmm.2013.1574 24317440

[B18] Hu Y. Chen H.-Y. Yu C.-Y. Xu J. Wang J.-L. Qian J. (2014). A long non-coding RNA signature to improve prognosis prediction of colorectal cancer. *Oncotarget* 5 2230–2242. 10.18632/oncotarget.1895 24809982 PMC4039159

[B19] Iyer M. K. Niknafs Y. S. Malik R. Singhal U. Sahu A. Hosono Y. (2015). The landscape of long noncoding RNAs in the human transcriptome. *Nat. Genet.* 47 199–208. 25599403 10.1038/ng.3192 PMC4417758

[B20] Jiang Q. H. Wang G. H. Jin S. L. Li Y. Wang Y. D. (2013). Predicting human microRNA-disease associations based on support vector machine. *Int. J. Data Min. Bioinform.* 8 282–293. 10.1504/Ijdmb.2013.056078 24417022

[B21] Ju J. Wang N. Wang X. Chen F. (2015). A novel all-trans retinoic acid derivative inhibits proliferation and induces differentiation of human gastric carcinoma xenografts via up-regulating retinoic acid receptor β. *Am. J. Transl. Res.* 7 856–865. 26175847 PMC4494137

[B22] Kohl M. Wiese S. Warscheid B. (2011). *Cytoscape: Software for Visualization and Analysis of Biological Networks Data Mining in Proteomics.* Berlin:Springer, 291–303. 10.1007/978-1-60761-987-1_18 21063955

[B23] Kong L. Zhang Y. Ye Z.-Q. Liu X.-Q. Zhao S.-Q. Wei L. (2007). CPC: assess the protein-coding potential of transcripts using sequence features and support vector machine. *Nucleic Acids Res.* 35(Suppl._2), W345–W349. 17631615 10.1093/nar/gkm391 PMC1933232

[B24] Kong R. Zhang E.-B. Yin D.-D. You L.-H. Xu T.-P. Chen W.-M. (2015). Long noncoding RNA PVT1 indicates a poor prognosis of gastric cancer and promotes cell proliferation through epigenetically regulating p15 and p16. *Mol. Cancer* 14:82. 10.1186/s12943-015-0355-8 PMC4399399 25890171

[B25] Langfelder P. Horvath S. (2008). WGCNA: an R package for weighted correlation network analysis. *BMC Bioinformatics* 9:559. 10.1186/1471-2105-9-559 19114008 PMC2631488

[B26] Lawrence M. Huber W. Pages H. Aboyoun P. Carlson M. Gentleman R. (2013). Software for computing and annotating genomic ranges. *PLoS Comput. Biol.* 9:e1003118. 10.1371/journal.pcbi.1003118 23950696 PMC3738458

[B27] Lazãr D. Tãban S. Raica M. Sporea I. Cornianu M. Goldiş A. (2008). Immunohistochemical evaluation of the tumor neoangiogenesis as a prognostic factor for gastric cancers. *Rom. J. Morphol. Embryol.* 49 137–148. 18516318

[B28] Li C. Liang G. Yao W. Sui J. Shen X. Zhang Y. (2016). Differential expression profiles of long non-coding RNAs reveal potential biomarkers for identification of human gastric cancer. *Oncol. Rep.* 35 1529–1540. 10.3892/or.2015.4531 26718650

[B29] Li H. Yu B. Li J. Su L. Yan M. Zhang J. (2015). Characterization of differentially expressed genes involved in pathways associated with gastric cancer. *PLoS One* 10:e0125013. 10.1371/journal.pone.0125013 25928635 PMC4415781

[B30] Liao Q. Liu C. Yuan X. Kang S. Miao R. Xiao H. (2011). Large-scale prediction of long non-coding RNA functions in a coding–non-coding gene co-expression network. *Nucleic Acids Res.* 39 3864–3878. 10.1093/nar/gkq1348 21247874 PMC3089475

[B31] Liu X.-H. Sun M. Nie F.-Q. Ge Y.-B. Zhang E.-B. Yin D.-D. (2014). Lnc RNA HOTAIR functions as a competing endogenous RNA to regulate HER2 expression by sponging miR-331-3p in gastric cancer. *Mol. Cancer* 13:92. 10.1186/1476-4598-13-92 24775712 PMC4021402

[B32] Liu Y. Sun M. Xia R. Zhang E. Liu X. Zhang Z. (2015). LincHOTAIR epigenetically silences miR34a by binding to PRC2 to promote the epithelial-to-mesenchymal transition in human gastric cancer. *Cell Death Dis.* 6:e1802. 10.1038/cddis.2015.150 26136075 PMC4650715

[B33] Liu Z.-M. Wang K.-P. Ma J. Zheng S. G. (2015). The role of all-trans retinoic acid in the biology of Foxp3+ regulatory T cells. *Cell. Mol. Immunol.* 12 553–557. 10.1038/cmi.2014.133 25640656 PMC4579645

[B34] Love M. I. Huber W. Anders S. (2014). Moderated estimation of fold change and dispersion for RNA-seq data with DESeq2. *Genome Biol.* 15:550. 10.1186/s13059-014-0550-8 PMC4302049 25516281

[B35] McKenna N. J. (2012). EMBO Retinoids 2011: Mechanisms, biology and pathology of signaling by retinoic acid and retinoic acid receptors. *Nucl. Recept Signal* 10:10003. 10.1621/nrs.10003 PMC3309077 22438793

[B36] Mermel C. H. Schumacher S. E. Hill B. Meyerson M. L. Beroukhim R. Getz G. (2011). GISTIC2. 0 facilitates sensitive and confident localization of the targets of focal somatic copy-number alteration in human cancers. *Genome Biol.* 12:R41. 10.1186/gb-2011-12-4-r41 PMC3218867 21527027

[B37] Morris T. J. Butcher L. M. Feber A. Teschendorff A. E. Chakravarthy A. R. Wojdacz T. K. (2013). ChAMP: 450k chip analysis methylation pipeline. *Bioinformatics* 30 428–430. 10.1093/bioinformatics/btt684 24336642 PMC3904520

[B38] Nguyen P. H. Giraud J. Staedel C. Chambonnier L. Dubus P. Chevret E. (2016). All-trans retinoic acid targets gastric cancer stem cells and inhibits patient-derived gastric carcinoma tumor growth. *Oncogene* 35 5619–5628. 10.1038/onc.2016.87 27157616

[B39] Ooi W. F. Xing M. Xu C. Yao X. Ramlee M. K. Lim M. C. (2016). Epigenomic profiling of primary gastric adenocarcinoma reveals super-enhancer heterogeneity. *Nat. Commun.* 7:12983. 10.1038/ncomms12983 PMC5052795 27677335

[B40] Patrad E. Niapour A. Farassati F. Amani M. (2018). Combination treatment of all-trans retinoic acid (ATRA) and γ-secretase inhibitor (DAPT) cause growth inhibition and apoptosis induction in the human gastric cancer cell line. *Cytotechnology* 70 865–877. 10.1007/s10616-018-0199-3 29417442 PMC5851978

[B41] Poulain S. Lacomme S. Battaglia-Hsu S.-F. Du Manoir S. Brochin L. Vignaud J.-M. (2009). Signalling with retinoids in the human lung: validation of new tools for the expression study of retinoid receptors. *BMC cancer* 9:423. 10.1186/1471-2407-9-423 19961602 PMC2797528

[B42] Qi R. Ma A. Ma Q. Zou Q. (2019). Clustering and classification methods for single-cell RNA-sequencing data. *Brief. Bioinform.* 4:bbz062 10.1093/bib/bbz062 31271412 PMC7444317

[B43] Ren W. Zhang J. Li W. Li Z. Hu S. Suo J. (2016). A tumor-specific prognostic long non-coding RNA signature in gastric cancer. *Med. Sci. Monit.* 22 3647–3657. 10.12659/msm.901190 27727196 PMC5072383

[B44] Shao Y. Ye M. Jiang X. Sun W. Ding X. Liu Z. (2014). Gastric juice long noncoding RNA used as a tumor marker for screening gastric cancer. *Cancer* 120 3320–3328. 10.1002/cncr.28882 24986041

[B45] Siegel R. Naishadham D. Jemal A. (2013). Cancer statistics, 2013. *CA Cancer J. Clin.* 63 11–30. 10.3322/caac.21166 23335087

[B46] Song H. Sun W. Ye G. Ding X. Liu Z. Zhang S. (2013). Long non-coding RNA expression profile in human gastric cancer and its clinical significances. *J. Transl. Med.* 11:225. 10.1186/1479-5876-11-225 24063685 PMC3848969

[B47] Stewart T. A. Yapa K. T. Monteith G. R. (2015). Altered calcium signaling in cancer cells. *Biochim. Biophys. Acta* 1848 2502–2511. 10.1016/j.bbamem.2014.08.016 25150047

[B48] Subramanian A. Tamayo P. Mootha V. K. Mukherjee S. Ebert B. L. Gillette M. A. (2005). Gene set enrichment analysis: a knowledge-based approach for interpreting genome-wide expression profiles. *Proc. Natl. Acad. Sci. U. S. A.* 102 15545–15550. 10.1073/pnas.0506580102 16199517 PMC1239896

[B49] Sun M. Jin F.-Y. Xia R. Kong R. Li J.-H. Xu T.-P. (2014). Decreased expression of long noncoding RNA GAS5 indicates a poor prognosis and promotes cell proliferation in gastric cancer. *BMC Cancer* 14:319. 10.1186/1471-2407-14-319 24884417 PMC4022532

[B50] Sun W. Wu Y. Yu X. Liu Y. Song H. Xia T. (2013). Decreased expression of long noncoding RNA AC096655. 1-002 in gastric cancer and its clinical significance. *Tumor Biol.* 34 2697–2701. 10.1007/s13277-013-0821-0 23645148

[B51] Sun W. Yang Y. Xu C. Xie Y. Guo J. (2016). Roles of long noncoding RNAs in gastric cancer and their clinical applications. *J. Cancer Res. Clin. Oncol.* 142 2231–2237. 10.1007/s00432-016-2183-7 27246953 PMC11819183

[B52] Tang W. Wan S. Yang Z. Teschendorff A. E. Zou Q. (2017). Tumor origin detection with tissue-specific miRNA and DNA methylation markers. *Bioinformatics* 34 398–406. 10.1093/bioinformatics/btx622 29028927

[B53] Tomczak K. Czerwińska P. Wiznerowicz M. (2015). The Cancer Genome Atlas (TCGA): an immeasurable source of knowledge. *Contemp. Oncol.* 19 A68–A77. 10.5114/wo.2014.47136 PMC4322527 25691825

[B54] Trapnell C. Pachter L. Salzberg S. L. (2009). TopHat: discovering splice junctions with RNA-Seq. *Bioinformatics* 25 1105–1111. 10.1093/bioinformatics/btp120 19289445 PMC2672628

[B55] Trapnell C. Williams B. A. Pertea G. Mortazavi A. Kwan G. van Baren M. J. (2010). Transcript assembly and quantification by RNA-Seq reveals unannotated transcripts and isoform switching during cell differentiation. *Nat. Biotechnol.* 28 511–515. 10.1038/nbt.1621 20436464 PMC3146043

[B56] Tsoi L. C. Iyer M. K. Stuart P. E. Swindell W. R. Gudjonsson J. E. Tejasvi T. (2015). Analysis of long non-coding RNAs highlights tissue-specific expression patterns and epigenetic profiles in normal and psoriatic skin. *Genome Biol.* 16:24. 10.1186/s13059-014-0570-4 PMC4311508 25723451

[B57] Wang G. Luo X. Wang J. Wan J. Xia S. Zhu H. (2018). MeDReaders: a database for transcription factors that bind to methylated DNA. *Nucleic Acids Res.* 46 D146–D151. 10.1093/nar/gkx1096 29145608 PMC5753207

[B58] Wang G. Wang Y. Feng W. Wang X. Yang J. Y. Zhao Y. (2008). Transcription factor and microRNA regulation in androgen-dependent and -independent prostate cancer cells. *BMC Genom.* 9(Suppl. 2):S22. 10.1186/1471-2164-9-S2-S22 18831788 PMC2559887

[B59] Wang G. Wang Y. Teng M. Zhang D. Li L. Liu Y. (2010). Signal transducers and activators of transcription-1 (STAT1) regulates microRNA transcription in interferon gamma-stimulated HeLa cells. *PLoS One* 5:e11794. 10.1371/journal.pone.0011794 20668688 PMC2909916

[B60] Yang F. Bi J. Xue X. Zheng L. Zhi K. Hua J. (2012). Up-regulated long non-coding RNA H19 contributes to proliferation of gastric cancer cells. *FEBS J.* 279 3159–3165. 10.1111/j.1742-4658.2012.08694.x 22776265

[B61] Yang Y. Chen L. Gu J. Zhang H. Yuan J. Lian Q. (2017). Recurrently deregulated lncRNAs in hepatocellular carcinoma. *Nat. Commun.* 8:14421. 10.1038/ncomms14421 PMC5316832 28194035

[B62] Yang Z. Guo X. Li G. Shi Y. Li L. (2016). Long noncoding RNAs as potential biomarkers in gastric cancer: opportunities and challenges. *Cancer Lett.* 371 62–70. 10.1016/j.canlet.2015.11.011 26577810

[B63] Yu G. Wang L.-G. He Q.-Y. (2015). ChIPseeker: an R/Bioconductor package for ChIP peak annotation, comparison and visualization. *Bioinformatics* 31 2382–2383. 10.1093/bioinformatics/btv145 25765347

[B64] Zeng X. Lin W. Guo M. Zou Q. (2017). A comprehensive overview and evaluation of circular RNA detection tools. *PLoS Comput. Biol.* 13:e1005420. 10.1371/journal.pcbi.1005420 28594838 PMC5466358

[B65] Zeng X. Liu L. Lü L. Zou Q. (2018). Prediction of potential disease-associated microRNAs using structural perturbation method. *Bioinformatics* 34 2425–2432. 10.1093/bioinformatics/bty112 29490018

[B66] Zeng X. Zhang X. Zou Q. (2015). Integrative approaches for predicting microRNA function and prioritizing disease-related microRNA using biological interaction networks. *Brief. Bioinform.* 17 193–203. 10.1093/bib/bbv033 26059461

[B67] Zhang B. Kirov S. Snoddy J. (2005). WebGestalt: an integrated system for exploring gene sets in various biological contexts. *Nucleic Acids Res.* 33(Suppl._2), W741–W748. 15980575 10.1093/nar/gki475 PMC1160236

[B68] Zhang W. Liu H. T. (2002). MAPK signal pathways in the regulation of cell proliferation in mammalian cells. *Cell Res.* 12 9–18. 10.1038/sj.cr.7290105 11942415

[B69] Zhao X. Jiao Q. Li H. Wu Y. Wang H. Huang S. (2020). ECFS-DEA: an ensemble classifier-based feature selection for differential expression analysis on expression profiles. *BMC Bioinform.* 21:43. 10.1186/s12859-020-3388-y 32024464 PMC7003361

[B70] Zhao Y. Wang F. Chen S. Wan J. Wang G. (2017). Methods of MicroRNA promoter prediction and transcription factor mediated regulatory network. *Biomed. Res. Int.* 2017:7049406. 10.1155/2017/7049406 28656148 PMC5474535

[B71] Zhao Y. Wang F. Juan L. (2015). MicroRNA promoter identification in arabidopsis using multiple histone markers. *Biomed. Res. Int.* 2015:861402. 10.1155/2015/861402 26425556 PMC4573627

[B72] Zou Q. Xing P. Wei L. Liu B. (2019). Gene2vec: gene subsequence embedding for prediction of mammalian N (6)-methyladenosine sites from mRNA. *Rna* 25 205–218. 10.1261/rna.069112.118 30425123 PMC6348985

